# Development and feasibility of a driving training program for Autistic student drivers

**DOI:** 10.1371/journal.pone.0324934

**Published:** 2025-06-27

**Authors:** Priscilla Vindin, Reinie Cordier, Nathan J. Wilson, Lauren Parsons, Hoe Lee

**Affiliations:** 1 Curtin School of Allied Health, Faculty of Health Sciences, Curtin University, Perth, Australia; 2 School of Arts and Humanities, Edith Cowan University, Joondalup, Australia; 3 Department of Social Work, Education and Community Wellbeing, Northumbria University, Newcastle upon Tyne, United Kingdom; 4 Department of Health & Rehabilitation Sciences, Faculty of Health Sciences, University of Cape Town, Cape Town, South Africa; 5 School of Nursing and Midwifery, Western Sydney University, Hawkesbury, Australia; 6 Department of Rehabilitation Sciences, Hong Kong Polytechnic University, Kowloon, HKSAR, China; Universitat de Girona, SPAIN

## Abstract

Driving licencing rates remain lower for autistic individuals capable of driving a motor vehicle, which can limit achieving independence in community mobility. However, there is limited autism-specific guidance in current driver training. The development and evaluation of the feasibility of an autism-specific Driving Training Program (DTP) intervention was conducted to improve the likelihood that autistic student drivers will safely and successfully learn to drive a motor vehicle and gain a driver’s licence. The DTP intervention was developed using a modified stepped approach for developing complex skills-based interventions. The Goals for Driving Education framework for explaining driving training behaviour modification formed the foundation of the intervention. A small-scale study was conducted using a single group pre-post-test design (*n* = 5), followed by semi-structured interviews and a survey (*n* = 12) to evaluate the feasibility of intervention components and participant acceptability. The driving performance of the autistic student drivers significantly improved, demonstrating the feasibility of the DTP intervention for training autistic student drivers to learn to drive. Participants also found the intervention acceptable, with program component refinement suggested. The DTP intervention is feasible for a larger randomised controlled trial after modifying highlighted program components.

## Introduction

The rising global prevalence of autism is leading to increasing numbers of autistic people learning to drive a motor vehicle and gaining their driver’s licence. Although many autistic individuals are cognitively capable of learning to drive, licencing rates remain lower for these individuals compared to non-autistic peers [1]. Learning to drive and gaining a driver’s licence can be challenging for autistic individuals and fraught with barriers for families and professional driving instructors who support autistic student drivers [1]. Driving is a complex task, and autistic traits such as differences in social and communication skills, motor coordination, visual perception, and executive functions impact driving skill acquisition [2–4]. Furthermore, deciding whether an autistic person is fit to drive, what age to begin driving training, or who may be the best person to conduct driving lessons can also be challenging for families [1,5].

Driving education and training are typically designed for non-autistic student drivers without considering the support needs of autistic individuals [5]. Additionally, standard driving training does not equip professional driving instructors with knowledge of the impact autism can have on learning to drive or practical training strategies to use when it does [1]. Furthermore, guidelines on autism and driving and autism-specific on-road driver training programs are generally lacking [6,7]. Therefore, there is a need to develop and implement autism-specific driving interventions addressing these challenges and informing how to proceed through theoretical and practical driver education and training.

Driving is an instrumental activity of daily living; therefore, interventions need to focus on the specific skills required to develop the ability to drive a motor vehicle on-road [8,9]. Also, for autism-specific programs, person-environment fit needs to be considered by understanding the potential barriers, challenges, and behaviours of autistic student drivers within the context of driving training environments [10]. To this end, evidenced-based psychosocial interventions should: (a) engage intervention end-users [11]; (b) include multidisciplinary collaborations [12]; (c) be informed by theory [13]; and (d) evaluate feasibility, appropriateness, and effectiveness [14].

### Developing complex interventions

Complex interventions are used to address public health practices and services, such as improving community mobility for autistic people. Complex interventions target multiple end-users, follow multiple steps during development and include multiple interacting components to achieve various outcomes [12,13,15]. Guidelines on developing and evaluating complex interventions have been provided by the United Kingdom’s Medical Research Council (MRC) and are widely used as a comprehensive approach covering the development, feasibility, evaluation and implementation phases of non-pharmacological interventions [13,16,17].

Comprehensively describing intervention development and evaluating feasibility is essential for providing evidence-based guidance for addressing aspects of functioning. In relation to driving interventions for autistic people, research specifically focusing on the use of the evidenced-based driver education frameworks is needed to understand the interactions between autism and driving performance [18]. One such framework, the Goals for Driver Education (GDE) framework, is a theoretical model identifying factors to consider for effective driver education and training and has been recommended as a basis for developing autism-specific driver training [19]. Intervention development studies are therefore required to understand if the GDE framework has potential as a driving training method for autistic student drivers [17]. The importance of methodological rigour in the initial stage of intervention development is recognised yet this complex stage is often unpublished [20]. Documenting the development of a complex intervention is particularly critical due to the intricacy of the interacting components. Future intervention developers can learn from the processes and decisions of others, and critical active ingredients are made clear so as not to become weakened during processes of adaptation or implementation [20,21], ultimately strengthening the continuum from research to practice [9,15].

In addition, a taxonomy of intervention development approaches provides further key actions across domains, including conception and planning, designing and creating, refining, documenting and evaluation planning, to aid researchers’ decision-making process during the intervention development phase [15]. One of the key actions in the refining domain is to test the developed intervention on a small sample for feasibility and acceptability using mixed methods [15]. Modelling the intervention before piloting and full-scale evaluations of effectiveness is an essential step to developing complex interventions further as it provides opportunities to refine and modify intervention techniques, work out measures, and assess the preliminary significance of effects [9,13]. Further, the need for feasibility testing to demonstrate the intervention can be delivered as planned and is acceptable to end-users is emphasised [9,13,14].Therefore, applying intervention development guidelines and key actions, this paper focuses on describing the development of a novel autism-specific Driving Training Program (DTP) intervention, and evaluates its feasibility. The DTP intervention was intended to improve the likelihood of autistic student drivers safely and successfully learning to drive a motor vehicle and gain their driver’s licence through autism-specific driving education and training guidelines. Specifically, this paper aimed to: (a) describe the development of the DTP intervention; and (b) explore the feasibility of the intervention by testing preliminary effectiveness and assessing participants’ perceptions of the intervention.

## Methods

The development of the DTP intervention was conducted by a team of occupational therapy, psychology, and nursing researchers with extensive experience in the areas of autism, transportation, community mobility, and people with disabilities. To ensure the final iteration of a complex intervention is both effective and sustainable, and to increase the number of key actions taken [15], a careful approach to development must be adopted. Two intervention development approaches guided this study: The Six Steps for Quality Intervention Development [12] and Intervention Mapping [22]. This combination of approaches was adopted as both utilise multiple systematic steps for improving complex problems and are amenable for application to psychosocial contexts such as autism and driver education [9,15].

### Procedures

**Fig 1:**
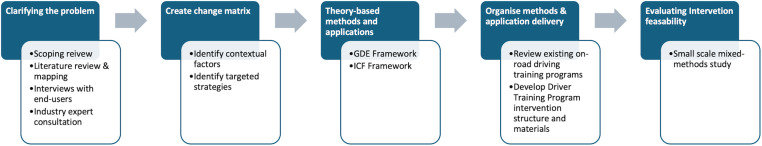
Intervention development process adopted for the study.

Both the Six Steps for Quality Intervention Development [12] and the Intervention Mapping [22] approaches include six iterative stages from initial development to evaluating effectiveness. As the focus of this study was the development and feasibility of a novel autism-specific DTP, five iterative stages were followed: 1) clarifying the problem, 2) creating a change matrix, 3) including theory-based methods and applications, 4) organising methods and applications delivery, and 5) evaluating the intervention feasibility. This paper does not report the final sixth stage (effectiveness). The stages of this process are detailed in Fig 1. The sixth stage, evaluating the effectiveness of the intervention for implementation, is reported in Vindin et al. [23].

### Clarifying the problem

A series of research activities and studies comprised this first stage of DTP intervention development. The research activities included a scoping review on autism and learning to drive [7], a literature review and mapping of individual and environmental contextual factors, strategies, and theory linked to driving training and autism, a qualitative study on the experience of learning to drive and autism [1], and consultation with industry experts.

The scoping review ascertained the extent of current evidence about autism and learning to drive. The findings indicated that autistic individuals drive differently from non-autistic peers, particularly in tactical driving skills (e.g., turning, overtaking, avoiding obstacles); however, the extent of these differences was not known. The scoping review also suggested tactical driving skills can be improved via training and highlighted the lack of autism-specific driving training programs [7]. Additional literature searches were conducted by the first author using five databases (CINAHL, Medline, ProQuest, PsycINFO, PubMed) to identify relevant literature related to: (a) the underlying contextual factors associated with autism that impact driving training and gaining a driver’s license, and (b) effective strategies for addressing the contextual factors for potential application in driving education and training. The first author reviewed all studies, followed by a review by the research team via planning meetings to determine factors and strategies for consideration when developing the change matrix.

Focus groups and individual interviews were conducted to gain the perspectives of the intervention end-users (i.e., autistic student drivers, their family members, and professional driving instructors). Barriers and challenges faced by autistic student drivers when learning to drive were identified, such as the complexity associated with autism and driving (e.g., extreme anxiety, motor coordination challenges, and difficulties with social communication in driving contexts) and external challenges to overcome (e.g., added time and costs required to learn to drive) [1]. Recommended strategies aimed at improving the success of driver education were also highlighted, and findings were used to develop guiding questions for the next research activity: consultation with industry experts.

To identify what was already known about the on-road driving behaviours of autistic student drivers and current strategies being successfully implemented during driving training, the first author consulted with two industry experts. One expert was a driver-trained occupational therapist and licensed driving instructor with driver assessment and rehabilitation expertise. The other was a qualified rehabilitation driving trainer with extensive experience working with autistic student drivers and autism lived experience. Both experts were interviewed, and one provided a report covering topics such as driving and anxiety (i.e., pre-lesson, in-car, and after obtaining a license) and in-car instruction (i.e., instruction choice, teaching techniques, progression, and discontinuation of lessons). The experts confirmed the autism-specific contextual factors impacting driving training ascertained from the literature review and provided additional driving training strategies to include in the DTP intervention, such as the use of cue words and a learner driver profile. The information gained from this series of research activities was then used in subsequent stages of the intervention development process.

### Create a change matrix

Based on the evidence gathered in the previous stage, a change matrix was developed, aligned with the intended goal of the intervention to improve the success of autistic student drivers learning to drive safely. There were two parts to the change matrix: (a) identifying the underlying contextual factors associated with autism impacting driving abilities to be targeted during driving training, and (b) aligning strategies to address the contextual factors for autism-specific driving training.

The research team reviewed 18 contextual factors identified in the previous step. The 18 factors were collapsed into the following eight contextual factors as areas of focus for the autism-specific DTP intervention: anxiety, executive functions (e.g., working memory, planning and organising, and impulse control), attention, cognitive rigidity (e.g., perfectionism), hazard perception (e.g., visual scanning and situational awareness), sensory processing (e.g., sensory overload), motor coordination, and social communication. While some of these contextual factors overlap, the strategies garnered during the previous step informed the decision to designate this particular set of eight. For example, although attention is an executive function, the magnitude of impact on driving associated with attention difficulties for autistic individuals meant that specific targeted strategies for attention during training were required. Similarly, hazard perception encompasses sensory processing, executive functions, and social communication, but was targeted as a stand-alone factor based on the magnitude of impact associated with hazard perception difference for autistic drivers. The eight contextual factors were aligned with potential strategies from the research that could aid driving training to accomplish the intervention goal. For example, the contextual factor of attention aligned with driving training strategies, thereby advising driving lessons to be conducted without passengers in the car to reduce distraction.

### Include theory-based methods and applications

Next, evidence-based theoretical methods and strategies to inform autism-specific driving training were selected. The GDE framework and the International Classification of Functioning, Disability and Health (ICF) were chosen and integrated with the change matrix to guide the organising structure of the intervention components.

### Driving theory and autism

The GDE framework is informed by traffic psychology research and explains behaviour modification concerning driver education and training [8,24]. Underpinned by cognitive psychology, the GDE emphasises individual motivations and pre-existing mental schemas as critical to behaviour modification. The GDE framework uses a constructivist pedagogical approach to aid the development of DTP intervention content, acknowledging both the driving instructors’ and student drivers’ knowledge [8].

The GDE framework is hierarchical, composed of two dimensions: (a) four levels of driver behaviour and goals, and (b) three essential content categories for driver education. The four levels of driver behaviour and goals range from concrete to general to abstract and include operational (e.g., steering, braking), tactical (manoeuvring in response to circumstances of the trip), strategic (e.g., planning of the trip), and behavioural aspects of driving [25,26]. Although the hierarchy levels are qualitatively different, they are interactive, with each level affecting the whole system. Therefore, higher-level abstract behavioural goals (e.g., lifestyle goals) impact lower-level concrete tactical goals (e.g., vehicle control) and vice versa [8,24].

The three content categories important for student driver training are: (a) basic knowledge and skills, (b) knowledge and skills related to risk-increasing factors, and (c) self-evaluation and awareness skills, and should be considered at each level of the driver behaviour and goals hierarchy [8,24]. Peräaho and colleagues distinguish between training skills and training student drivers to become aware of potential risk-increasing factors impacting their driving knowledge and skills [8]. Self-evaluation and awareness skills are essential tools during student driver training and for ongoing driving experience after learning to drive. Hatakka and colleagues point out that self-evaluative skills do not develop automatically [24]; therefore, educational strategies for increasing student drivers’ awareness of personal tendencies must be included in driving training.

A description of the alignment between selected examples of autism-specific contextual factors associated with driving (identified in the previous stage) and the dimensions of the GDE framework for driver education and training are described below in Table 1. An explanation of the different GDE levels as applied to the study context is provided forthwith.

**Table 1:** Alignment of GDE framework with examples of autism-specific contextual factors associated with driving.

		Essential Content Elements of Driver Training		
		Knowledge and skills to master	Awareness of risk-increasing factors	Self-evaluation and awareness skills
**Hierarchical levels of driver behaviour**	**Level 4: Goals for life and skills for living**	Age/Gender Fitness to drive Motivation to drive	Hazard perception: personal motives and behavioural tendencies Anxiety	Anxiety impacts and emotion regulation skills Attention issues
	**Level 3: Goals and context of driving**	Planning abilities (e.g., evaluating weather conditions for a trip) Choice of driving instructor (e.g., parent or professional)	Hazard perception: trip-related decisions Time of day (e.g., fatigue, peak hour)	Decision-making and problem-solving skills Avoidance compensatory behaviours
	**Level 2: Mastery of traffic situations**	Decision-making (e.g., whether to pass a car) Multiperceptual driving skill difficulties	Hazard perception: traffic-related Social communication in the traffic environment	Strengths (e.g., road rules knowledge) Social communication difficulties
	**Level 1: Vehicle maneuvering**	Car control (e.g., control of driving position and direction) Visual perceptual differences	Hazard perception: skills related Motor coordination issues (e.g., psychomotor skills)	Level of confidence (high/low) Need for visual demonstrations

The highest level of the hierarchy, Goals for life and skills for living (Level 4), focuses on personal behavioural styles, motives, and goals. It suggests that behaviour modification required for driver training is impossible without considering the student driver’s personal goals and lifestyle factors [24]. This level includes consideration of preconditions, such as autism, which influence student drivers’ behaviours, attitudes, and motivations. For example, if an autistic student driver does not value driving as an important goal or if they are learning to drive because of external pressure, then lower levels of the hierarchy will be adversely impacted in the driving training process [1].

At this level of knowledge and skills, driving training strategies need to show student drivers that driving a vehicle is a behaviour that is impacted by their personal lifestyle, tendencies, and characteristics and that these behavioural factors will impact the choices they make at each level of the hierarchy when driving. Therefore, knowledge about the potential impacts of autism on driver behaviour is essential for driving education and training [8]. For example, the age at which some autistic student drivers commence driving lessons may need to be delayed from 16 to at least 23 years old to increase the chance of driving training success [1,27]. Also, an autistic student driver’s gender may influence their preference for a driving instructor of a particular gender due to social anxiety. Not being aware of the student driver’s fitness to drive or personal preferences could impact their motivation to attend driving lessons with a driving instructor [1]. To avoid the potential risk-increasing effects related to hazard perception issues, such as being slower to respond to hazards [4], autistic student drivers must be aware of how autism and hazard perception are interrelated. Targeted training strategies allowing student drivers to self-evaluate via reflection on how they personally engage with hazards, such as whether they avoid looking at pedestrians due to increased anxiety, need to be aimed at increasing awareness of how this tendency will impact their driving behaviour [1,4,8].

The second highest level of the hierarchy, Goals and context of driving (Level 3), focuses on the goals and context of driving and the decisions a driver makes concerning the driving trip, such as the purpose of the trip [8,24]. Decisions made at this level have important implications for on-road safety and are directly influenced by preconditions such as autism. For example, if an autistic student driver experiences regular fatigue early in the day, the decision to drive in the morning could impact how they respond while driving during a trip in peak-hour traffic [1]. Therefore, driving training needs to consider driving goals and contexts that may be affected by autism-specific factors.

Driver training knowledge and skills at this level will need to consider the autistic student driver’s cognitive processing needs, which may impact trip contexts, such as the level of support required to plan driving trips [4]. For example, autistic student drivers who are challenged by local processing (e.g., central coherence) and cognitive flexibility may face trip-related risk-increasing factors that impact hazard perception. Knowledge is required to target training strategies aimed at increasing student driver’s subjective awareness of any challenges they may face with decision-making when alternative decisions need to be made during a drive, such as responding to unexpected road works [1,4,8].

Next, Mastery of traffic situations (Level 2), focuses on knowing how to negotiate traffic situations. At this level, the driver has to adapt their behaviour in constantly changing traffic environments, such as negotiating changing road rules, hazards, and other road users’ behaviours [24]. The ability to negotiate traffic environments develops via on-road driving experience and is impacted by preconditions such as autism. For example, if an autistic student driver is challenged by making eye contact with other road-users due to social communication difficulties, their ability to predict other driver’s behaviours or make their own behaviours predictable to other drivers could be impacted [1,8,24].

Knowledge and skills training at this level needs to account for potential difficulties autistic student drivers experience with multi-perceptual driving skills, such as negotiating speed regulation, lane management, roundabouts, and parking [1,8]. For example, differences in cognitive flexibility may impact tactical skills needed when making decisions in traffic, such as knowing when to pass another vehicle or adapting speed in different driving environments [1,4]. Examples of traffic-related risk-increasing factors linked to hazard perception for autistic student drivers are differences in social communication and sensory processing challenges, which are especially important to focus on during driving training. Self-evaluation strategies at this level need to include awareness of personal skills in managing social communication and information overload in busy traffic environments, as well as awareness of strengths, such as having excellent knowledge of road rules [1,4,8].

The final level in the hierarchy, Vehicle maneuvering (Level 1), focuses on vehicle operations and interactions between student drivers and vehicle controls. Basic maneuvers required to operate a vehicle may need to be automated to avoid overwhelming information processing capacity needed to respond to other road-users’ behaviours [24]. Furthermore, preconditions such as autism, regulated by higher levels of the hierarchy, directly impact fundamental psychomotor skills required to operate and control a vehicle in traffic [4,28]. For example, suppose an autistic student driver experiences motor coordination challenges when steering a vehicle while watching oncoming traffic. In that case, the potential to become overwhelmed by the level of information processing required to control vehicle maneuvers will increase [1].

The knowledge and skills required at this level involve training strategies that emphasise potential interactions between the autistic student driver, vehicle, and driving environment [8]. Complex driving operational skills that require kinesthetic awareness, such as controlling the direction of a vehicle and blind spot checks, may be challenging for autistic student drivers due to perceptual processing differences. Motor coordination and perceptual processing challenges are examples of risk-increasing vehicle maneuvering factors that are linked to hazard perception, impacting reaction times and visually checking of the driving environment [1,4,8]. Self-evaluation and awareness of skills training need to emphasise any potential risks associated with an autistic student driver’s personal behaviours and unique challenges, such as how their level of driving confidence (low to high) may be problematic when operating a vehicle [1,8].

### Aligning theory

Next, the International Classification of Functioning, Disability and Health (ICF) framework [29] was aligned with the integrated GDE framework. To develop the DTP intervention, the ICF was used as a bridging theory aiding the description of autistic traits, strengths and challenges (i.e., contextual factors) as well as aligning with concepts within the GDE hierarchical levels for driving education and training [30,31]. The ICF is a bio-psycho-social framework based on a combination of medical and social health views, designed to establish a common language for describing health conditions such as autism [29,30] and can be used to define interventions for driving rehabilitation [31].

The ICF organises components into two parts: (a) functioning, which describes body functions and structures, activities, and participation, and (b) disability, which describes impairments, activity limitations, and participation restrictions. The ICF also incorporates personal and environmental contextual factors that interact with all functioning and disability components [29]. Combining the ICF components with the GDE allowed for problems with functioning associated with autism and driving to be clarified and structured into one organising framework. For example, autism impacts a person’s driving behaviours (*activities and participation*), which are directly influenced by impairments in body functions and structures (*disability*), such as impaired psychomotor, perceptual and attention functions, and, in turn, is influenced by the driving environment (*environmental contextual factors*), such as driving conditions, driving demands, the vehicle, and the driving teacher [30,31]. The ICF guided the translation of theory into practical strategies, informing the structure of the DTP intervention components.

### Organise program methods and application delivery

This stage involved combining the outcomes of the previous three stages to produce the DTP intervention [12,22]. As no existing on-road driving training programs were designed for autistic individuals [7], two resources were utilised to guide the intervention structure and practical delivery: The TeenDrivingPlan (TDP; [32]) and the Teaching Learner Drivers with Disabilities: An Operation Manual for Driving Instructors [33]. The TDP is a psychoeducational driving practice intervention for parents training non-autistic teens to drive and provides an organising structure for the DTP intervention. The TDP comprises three core components: (a) short educational videos, (b) practice drive planning tools to identify specific skills in the practice environments, and (c) interactive logging and rating tools to track practice and identify areas for further skill development. The TDP focuses on providing practice diversity, such as varying driving conditions (e.g., poor weather and night driving) to increase on-road driving safety and maximising practice hours across multiple driving contexts to develop safe, skilled drivers [32,34]. The Teaching Learner Drivers with Disabilities: An Operation Manual for Driving Instructors [33] highlighted modifications that need to be made to driving education approaches to accommodate for factors requiring additional support, such as motor coordination or visual scanning. This guide also provided a model for describing intervention strategies and adapting driving training for professional driving instructors [9,33].

### Evaluating the intervention feasibility

Consistent with the adopted intervention development approaches, a small-scale, mixed methods study was conducted to evaluate the intervention feasibility for refinement prior to the larger-scale trial [9,13,15]. This approach for conducting a small-scale feasibility study is aligned with the United Kingdom Medical Research Council’s framework for developing complex interventions that underscores the importance of feasibility testing, despite being overlooked or rushed in the past [13,17]. Feasibility was concerned with the preliminary effectiveness of the intervention and understanding whether the DTP intervention and study procedures were viable for end-users and conceptualised according to criteria outlined by Bowen and colleagues [35]: (a) *Acceptability*: participant reactions to the intervention; (b) *Implementation and Practicality*: the extent to which the program components were used as planned and program logistics were delivered; (c) *Limited Efficacy Testing*: changes in confidence levels and overall benefits, and (d) *Adaption*: suggested improvements, and planned modifications for a larger scale study. Ethics approval was granted by the Curtin University Human Research Ethics Committee (Approval number: HRE2016−0103), which included approval of consent procedures.

### Participants

Participants were autistic student drivers, their caregivers, and professional driving instructors. Recruitment of autistic student drivers occurred via convenience sampling during February 2018, using posters and expressions of interest generated through community organisations and autism research events. The eligibility criteria for autistic drivers were: (a) self-reported diagnosis of autism without a cooccurring intellectual disability (verbally verified as being diagnosed by a qualified professional); (b) current valid learner’s permit; (c) meeting the driving legal visual acuity requirement of 6/12 m (20/40 ft) or better on the Meter 2000 Series Revised ETDRS chart [36]; and (d) capable of independently undertaking the intervention tasks (e.g., fill in a logbook). Participants were excluded if they held a provisional or full driver’s licence or had previous driving lessons with a professional driving instructor. Driving instructors holding a current driving instructor licence were also recruited from one driving school to train the student drivers using the DTP intervention. Written consent was gained for all participants, and for the student drivers aged below 18 years, written consent was also obtained from parents. All participants in this study were determined to have the capacity to provide consent as they were attending mainstream secondary or post-secondary education, did not have an intellectual disability, and had successfully met the criteria to obtain a learner’s permit.

### Data collection instruments

A series of psychological measures were administered to establish a profile of participants and to evaluate the practicality of administering multiple assessments in a larger-scale study. The Driving Performance Checklist (DPC) was administered to evaluate *driving performance* as the outcome of interest. An interview guide and Likert scale questionnaire were developed to evaluate the feasibility of the DTP and the study from the participants’ perspective. Details on the data collection instruments is provided in S1 File.

### Procedure

Driving instructors participated in the 2-hour driver instructor training session conducted by the research team. Study information sheets, consent forms, and the battery of screening measures were sent to student drivers and their parents to complete. Researchers collected the measures and forms during an initial meeting where student drivers were screened for visual acuity and assigned a driving instructor. The student drivers were then asked to commence ten driving lessons with the DTP-trained driving instructors in an automatic transmission dual-controlled car provided by the instructors.

Two observation drives were conducted to measure the student driver’s on-road driving performance using the DPC at the beginning and end of the ten lessons. The first (pre-test) observation drives were conducted after initial driving lessons (mean lessons = 3) to maintain the safety of student drivers and ensure they were competent to complete on-road driving tasks. After completing all ten driving lessons, the second (post-test) observation drive was conducted. The drives lasted approximately 30 minutes, following a standardised driving route.

Semi-structured interviews were conducted with student drivers and parents after their ten lessons were completed and with driving instructors at the end of the study. Interviews were conducted by the first author, in person or by phone, for those unable to attend in person (e.g., due to travel distance). Interviews lasted 10–40 minutes, were audio-recorded, and transcribed verbatim by a professional transcription service.

### Data analysis

Statistical analysis was conducted using the Statistical Package for Social Sciences (SPSS version 25; [37]). As this was a feasibility test of the intervention, the study was not powered to evaluate intervention effectiveness. Rather, analysis was conducted to evaluate the preliminary effects of the intervention on a small sample and the utility of the outcome measure effects [9,13]. Descriptive statistics were calculated for DPC scores at both time points, and the Wilcoxon signed-rank test was used to assess whether DPC scores changed between pre- and post-DTP participation. Significance was set at *p* < 0.05. Effect size (*r*) was calculated and interpreted as follows: < 0.1 = no effect, 0 = small effect, 0.3 = medium effect, 0.5 = large effect [38].

A constant comparative analysis approach was used to analyse the interviews [39], and participant responses were mapped against four feasibility domains: acceptability, implementation and practicality, adaption, and limited efficacy testing. Gendered pronouns have been modified, and pseudo-names have been used to ensure participant anonymity. Medians were calculated per participant group for student driver and parent 10-point scale responses. Due to the small sample size, group-level results for driving instructors were not calculated.

## Results

### The intervention: Driving Training Program (DTP)

After completing the first four stages of the methods (i.e., clarifying the problem, creating a change matrix, including theory-based methods and applications, and organising methods and application delivery), the development of the DTP was complete. The DTP was designed to be delivered over 10 weeks during driving training sessions (approximately 2 hours per week) and included four components: (a) the DTP training manual, (b) learning log booklet, (c) training videos, and (d) driving instructor information session. Intervention resources were made available to all participants (i.e., student drivers, parents, and driving instructors). They included hard copies of the manual and learning log, and videos on a USB memory stick. The ten weekly driving training sessions were designed to be completed by the student driver and driving instructor. They included a driving lesson (approximately 1 hour), along with activities such as questionnaires, a learning log or watching videos. The term driving instructor is used throughout the intervention to describe a licensed adult supervising a student as they learn to drive, and it may include a family member, friend, community worker, or professional driving instructor.

**Fig 2:**
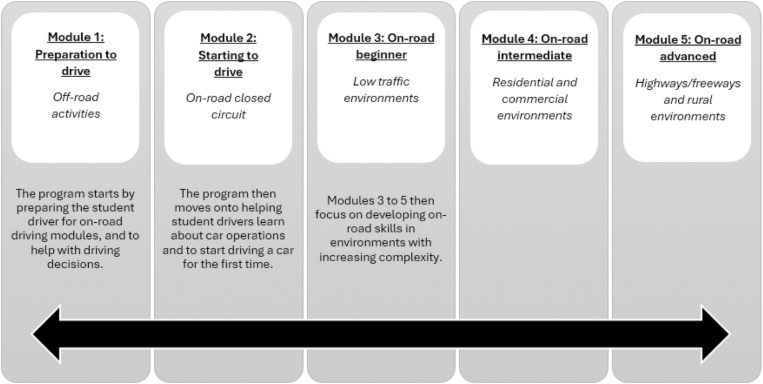
DTP Intervention manual modules.

*The DTP training manual* included a comprehensive guide on autism and driving to aid in understanding the support needs of autistic drivers when learning to drive. The manual introduction provided: (a) information on fitness to drive, indicators to discontinue lessons, identifying difficulties impacting driving, and practical driving assessment strategies; (b) links to state and territory driving handbooks and regulations on disclosure of autism; and (c) a manual user guide. The manual then included five graded modules covering the development of driving skills. Fig 2 represents the structure of the modules showing that during each driving lesson, instructors might incorporate activities from various modules.

Each module featured a range of structured activities designed to assist student drivers and driving instructors from preparing to drive and the first on-road lesson through to more advanced lessons. To ensure students could sufficiently practice driving skills, module activities were designed to be completed over a range of environments, such as car parks and residential areas. The activities followed a structured format, including an observation drive (e.g., task demonstration), activity details, goal, cue words, task (i.e., a description outlining the structure of the lesson activity), identifying issues, task mastery, and helpful information and tips. The activities were designed to be delivered at each student’s skill level, therefore catering to students with no on-road driving experience or who had already commenced driving lessons. The activities were designed to allow students to work through tasks at their own pace, developing competency of each skill before moving on to another task. Some students may have completed more than one task in a lesson, while others may have taken multiple lessons to become competent at a skill.

Each on-road module (modules 2–5) also began with targeted information sections for the student drivers and driving instructors. Students were asked to complete self-reflection activities consisting of a series of questions, 5-point confidence rating questions assessing confidence levels (e.g., how confident do I feel about driving a car in a quiet area?), and responses to personal reflection questions (e.g., what are the reasons that I decided to learn to drive a car? What driving conditions do I think are risky?). Driving instructors were encouraged to review student’s answers when commencing lessons in a new module. The questions were aligned with the GDE levels and content categories for driving training to provide greater awareness of students risk-increasing factors as well as the opportunity to improve their self-evaluation skills and receive feedback [8,24]. Driving instructors were also encouraged to read autism-specific information (e.g., car modifications such as blind spot mirrors or a digital speedometer that augment the sensory environment, or anxiety management strategies). The instructor’s information included a series of questions aligned with each driving environment (e.g., does the student driver experience increased anxiety as the driving demands increase?). The purpose of the instructor section was to help build a productive learning environment, recognise autism-specific challenges and barriers when learning to drive, and provide evidence-based strategies for responding appropriately. Parents were also encouraged to read the manual to adequately support students, communicate with instructors, and assist in implementing the DTP intervention during driving lessons.

*The DTP learning log* was a companion workbook to the manual, designed to assist student drivers and driving instructors in planning lessons, monitoring progress, and recording task acquisition. The student was responsible for using the learning log before, during, and after each lesson. The instructor could use the learning log to assign homework tasks, provide written feedback, communicate with family members or caregivers, and rate the level of task competence after each lesson. The learning log comprises four sections: (1) a personal profile, including general demographic information, previous driving experience, health information, sensory processing information, and abilities and interests; (2) learning styles information, based on Gardner’s [40] theory of eight multiple-intelligences, aligned with identifying signs and tips to help in driving lesson contexts; (3) a modified four-point task competence rating tool adapted from the TDP [32,41] aligned with activities in the manual modules, and (4) a learning log for each lesson recording the date, time and length of the lesson, location, environment conditions (e.g., raining), lesson goal (e.g., drive for 20 minutes), students reflection or evaluation of lesson goal, homework task (e.g., watch roundabout video), and driving instructor feedback. Sections one and two of the learning log were completed by the student, three by the instructor, and four by the instructor and student.

The purpose of the learning log was to help reduce anxiety for students by providing opportunities to plan and prepare for lesson expectations as well as facilitating rapport building between students and instructors. The personal profile provided information unique to each student. For example, if a student was prone to sensory overload, the instructor had knowledge of person-specific triggers and strategies. Furthermore, this section included the student’s interests and hobbies, which could be used as discussion topics to help instructors build rapport or as a management strategy, such as distracting the student during increased anxiety situations. The learning styles information assisted in lesson planning by matching students’ unique learning needs to training strategies and as a management tool. For example, if a student indicated they respond well to getting instructions graphically, the instructor could use visual diagrams of traffic scenarios drawn on a whiteboard. Students were also encouraged to record their reflections on lesson goals to discuss with instructors, supporting awareness of risk-increasing factors and self-reflection skills. Parents were also encouraged to read the learning log to keep track of their child’s progress and respond to strengths and challenges.

*Driving training videos* included ten pre-existing supplementary driving instruction training videos aligned with activities within the manual modules. The videos covered strategies and visual demonstrations of driving skills, such as looking for hazards, overtaking and keeping distance. Student drivers were instructed to watch the videos before lessons covering activities such as ‘intersections’ [42].

*Driving instructor training* consisted of a 2-hour, in-person information session designed for driving instructors to complete before implementing the DTP. The training sessions focussed on preparing the driving instructors recruited to conduct the DTP intervention during the feasibility study. The driving instructor information session included two components: (a) *Autism and Driving*, which covered information on autism and specific barriers and challenges faced by autistic student drivers, and (b) the *DTP Intervention*, which covered the use of the training manual, the driving instructor’s role, and lesson planning examples.

### Evaluating feasibility

#### Participants.

Five autistic student drivers (male *n* = 4) aged 16–19 years (mean = 17.2 years, see Table 2) and one parent of each student driver (mothers, *n* = 5) took part in the study. Two professional driving instructors were also recruited.

**Table 2:** Student driver demographics.

Driver demographic variables	
Mean age in years (range)	17.2 (16-19)
Gender (male)	4 of 5
Place of birth (Australia)	4 of 5
Current education participation	
High school (Yr 12)	4 of 5
TAFE^a^	1 of 5
Employed	1 of 5
Reside (home)	5 of 5
Previous driving experience (yes)	3 of 5
Mean length of experience	
Months (range)	3 (0–8)
Driving hours (range)	10.2 (0–27)
Glasses (yes)	1 of 5
Mean scores on screening measures (SD)	
BRIEF-A (informant report)	64.60 (9.21)
BRIEF-A (self-report)	67.60 (9.81)
CANTAB	9440.60 (4802.46)
RCMAS-2	57 (9.93)
SRS-2	75.60 (4.88)
BIS-11	65.40 (9.66)
ADSES	46.20 (7.79)

^a^ TAFE = Technical and Further Education; SD = standard deviation. Executive function measured by BRIEF-A and CANTAB; anxiety measured by RCMAS-2; social responsiveness measured by SRS-2 (adult version); Impulsiveness measured by BIS-11; driving self-efficacy measured by ADSES. Total or composite scores reported (see S1 File).

### Preliminary effectiveness

All student drivers showed an increase in DPC scores from pre- to post-observation drive, with the median DPC score increasing from 124 (IQR = 96–133.5) to 139 (IQR = 134–141.5). The Wilcoxon signed-rank test showed a statistically significant increase in driving performance for autistic student drivers (*z *= −2.023, *p* = 0.043), with a large effect size (*r* = .90).

### Participant perspectives

Findings from the constant comparative analysis of interviews are presented below. Descriptive statistics for 10-point Likert scale questions posed at the end of the interviews are presented in Table 3.

**Table 3:** Results of Likert-scale questions asked during interviews.

Items	Student Drivers (*n *= 5) Md (IQR)	Parents (*n *= 5) Md (IQR)
*Experience* ^a^		
Your experience doing the driving training lessons	8 (7-9)	
Your experience with the driving instructor	8 (7-9)	9 (5-10)
Your experience being involved in the driving training intervention		9 (7.5-10)
Your child’s experience with the driving instructor		10 (6-10)
Your experience conducting lessons using the intervention		
*Confidence* ^a^		
Your confidence in learning to drive	7 (5.5-7)	
Your confidence in gaining a driver’s licence	7 (6-7.5)	
Your child’s confidence in learning to drive		7 (7-9)
Your confidence in your child gaining their driver’s licence		9 (7.5-10)
*Benefits* ^a^		
Benefits to your driving skills in doing the training lessons	8 (6-9.5)	
Benefits to your child’s driving skills since completing the training lessons		8.5 (7-10)
Benefits to students driving skills in doing the intervention training lessons		
*Communication* ^a^		
Communication between you and the research team		10 (8.5-10)
Communication between you and the driving school/ instructor		8 (3-9)
*Overall Usability* ^b^		
Rate the DTP intervention usability	6 (4.25-9.25)	8 (3-10)^*^

*Notes.* Md = Median; IQR = Interquartile range.

^a^ 1-10 (I don’t agree at all – totally agree).

^b^ 1-10 (very easy – very difficult).

* Item missing 2 responses.

### Acceptability

All three participant groups had a positive reaction to the DTP intervention. All student drivers expressed the lessons were an effective way to learn to drive, and parents expressed their appreciation for being involved: “I thought it was just a fantastic opportunity”. They highlighted positives from the experience, such as gaining insight into their child’s capabilities, as expressed by one parent: “I just think it’s given me more clarity for Joseph and given more clarity for him to know that he can do this [learn to drive] and it’s just so positive”. The survey of their intervention experience supported these responses.

When the driving instructors were asked about their experiences conducting the driving lessons, they said their experiences were interesting and informative. One stated that it helped with driving lesson planning: “It was good, because I would write down what I needed to for the lesson that I’d just had with the student, and then I would be able to then go back to that on the next lesson to remind myself of what we needed to look at for this lesson”. However, the other driving instructor stated that they found the lesson structure rigid compared to their regular training approach: “I did find it probably more restrictive in the way that I teach.”

### Implementation and practicality

Sentiments about the utility of the DTP components were generally positive; however, not all intervention components were implemented as planned. When asked about using the manual, several student drivers indicated they read the information, although not often, with one student sharing, “I’ve been kind of busy with schoolwork, but yeah, I looked at it for a few times”. Parents explained getting their child to complete the logbook tasks was challenging, which was supported by one student driver who found “the actual writing” tasks difficult. Notably, only one driving instructor used the components as planned throughout the study, responding that “…the whole logbook concept is really good, and I think a lot of instructors are going to find it to be very helpful” and “…the front part [personal profile] where I learnt about the student was really good, because it taught me what I was to do with Shane if anything should go wrong”. In contrast, the other driving instructor did not use the manual consistently after the training session, responding, “For the most part, it was pretty much what I already knew, so it was just a quick scan for me”.

Generally, program logistics were delivered with all student drivers completing all ten driving lessons and both on-road observation drives. However, challenges associated with the operations of the driving school were identified, for example, issues with scheduling lessons with driving instructors were discussed: “There were a number of occasions where those lessons were rescheduled at the last minute, or they were forgotten about or re-arranged, or the time was wrong”.

### Limited efficacy testing

All participant groups identified benefits and changes for the student drivers following the intervention. One of the main benefits described was an increase in student driver confidence in learning to drive and driving on-road as described by one parent, “Confidence with people, cars, with roads, with situations he didn’t think he’d be able to handle, he proved to himself that he could”. One student driver responded, “I’ve gained a lot more confidence in my driving and driving on the road”. Several student drivers agreed that they benefited from a shift in their self-perceived driving capabilities, “Well, just inspiring me… I thought that I was a worse driver than I actually was”. Both driving instructors agreed the student drivers benefited from participating in the intervention, noting a decrease in student anxiety levels, which were high in the beginning lessons, “[Sarah] has gained a lot and is no longer anywhere near as anxious as she was about driving, and she has actually started to build up some excitement about actually being able to get out there and drive and get her licence”. Parents and driving instructors described noticing a change in motivation, willingness, and excitement to drive. This was especially important to parents who described their children’s low motivation to participate in driving lessons before the intervention. The survey ratings supported these responses when asked about confidence and benefits.

### Adaption

All participant groups suggested changes to the manual presentation, such as simplifying the text content, using dot points, and incorporating visual imagery, as supported by one driving instructor’s responses, “I’m not a reader, and when it comes to text, I’m a visual doing learner”. Participants also suggested converting from a paper-based format to a web-based platform with interactive components for the logbook and manual. As noted in one parent’s response, “Something that’s not writing... If it was an app, you’d have much better success... it would overcome that barrier of unwillingness [to write]”. These modifications were suggested to counter the challenge parents faced with getting their child to complete the written tasks, and driving instructors found the extra time required to record tasks restrictive, often impacting lesson time.

## Discussion

It is widely accepted that complex intervention development should be guided by systematic approaches, ensuring they are feasible and effective. This paper describes the development and feasibility of a novel autism-specific driver training intervention. Intervention development was guided by modified evidence and theory-based approaches, which proved appropriate frameworks for developing and evaluating this skills-based intervention. The DTP intervention was informed by research and grounded in theoretical frameworks: the GDE framework for behaviour modification related to driver education and training, integrated with the ICF. Using these theoretical frameworks allowed for targeted driving training strategies aimed at addressing the needs of autistic student drivers. A key aspect of the development process was the small-scale study, which evaluated the preliminary effectiveness and feasibility of the DTP intervention and refined it for larger-scale evaluation.

In this feasibility study, the driving performance of autistic student drivers following ten sessions of the DTP showed a significant increase, with a large effect size, suggesting that the DTP may be an effective approach to driver training for autistic student drivers. The DPC measure primarily evaluated aspects of driving related to GDE framework Levels 1 and 2 (i.e., vehicle manoeuvring and understanding of traffic situations), which are conceptually linked to the on-road driving elements of the manual. Driving instructors have described a need to scaffold instruction, use repetitious phrases or driving commentary, and individualise instructional strategies to promote practical driving skill development of autistic drivers [43,44]. The graded structure of the modules and the range of techniques related to the practical elements of driving, therefore, likely contributed to the learning of student drivers in this study. As this initial study was a feasibility study, assessing the efficacy of the DTP intervention was not the primary objective. The lack of a control group and small sample means this finding is not evidence for the effectiveness of the approach, nor does it provide evidence that any specific technique contributed to the effect measured.

From the perspective of parents and student drivers alike, a key aspect of acceptability was positive changes in their beliefs about driving, motivation to attend driving lessons, and increased confidence in learning to drive and gaining their driver’s licence. This increased motivation and confidence aligns with behavioural modification suggested in Level 4 of the GDE [8,24]. Autistic student drivers may not have the same intrinsic motivation to learn to drive as their non-autistic peers, and either high or low levels of confidence impact driving training [1]. Furthermore, driver education and training focus predominantly on operational and tactical skill development (i.e., GDE Levels 1–2) without considering behavioural factors influencing student drivers [8,24]. The DTP intervention included a deliberate emphasis on higher-level, abstract, contextual and behavioural factors via a focus on self-evaluation strategies and personalised programs to account for individual psychosocial differences [45–47]. Therefore, the focus on context-specific risks and self-evaluation strategies for autistic drivers within the DTP intervention suitably addresses higher levels of the GDE, contributing to the acceptability of the intervention for its end-users.

The delivery of the lessons by a professional driving instructor contributed to the practicality of the DTP for participants. One explanation for this finding is that parents are not experts in driving instruction, and parent anxiety and parent-child relationship dynamics can be barriers to the driving education process for autistic young people [1]. The cost of learning to drive via professional driving instruction can be prohibitive for some families; however, this study observed high lesson completion rates. Compliance with the ten-lesson protocol could be due to the subsidised cost of professional driving instruction delivered through research. Within the Australian context, autistic young people may be able to access financial support for driving instruction through the National Disability Insurance Scheme [NDIS; 48]. Given participants’ preference for the delivery of lessons by a professional driving instructor, understanding and consideration of the resources available to autistic young people to facilitate participation and community mobility need to be considered for future implementation of the DTP intervention.

While participants largely supported the feasibility of the intervention, they identified a need to modify the manual and logbook to a web-based platform with an emphasis on simplifying the presentation of written information and including schema to convey information. While an autism diagnosis is not predictive of reading comprehension challenges, reading comprehension is underpinned by oral language skills, and autistic individuals with co-occurring language difficulties likely require additional support for reading comprehension [49]. Implementing strategies to deliver key information to autistic student drivers to reduce reliance on reading comprehension will increase the accessibility of these intervention materials for all participants, regardless of reading comprehension ability. Delivering the DTP materials via a web-based platform also poses several potential advantages. Student drivers expressed a preference for online engagement with materials, which also presents many advantages over paper-based materials, including accessibility for all participant groups and larger numbers of participants, reducing the time to complete tasks, and the ability to measure engagement and monitor use [32,50]. These modifications are recommended for incorporation into future versions of the DTP intervention.

Additionally, findings suggest that lesson flexibility needs to be factored into future DTP development to account for the real-world context of driving training. Given that some student drivers will have no driving experience, while others will have some lessons with parents, finding a balance between uniformity and individual delivery needs to be factored into the intervention [9]. This can be achieved by clarifying the non-sequential delivery of modules and providing lesson planning examples during the driving instructor training sessions, which need to be included in future study procedures.

Variations in implementation fidelity by driving instructors were evident within this study, with mixed findings regarding the feasibility of the intervention components for driving instructors. Multi-faceted training approaches are likely required to achieve behaviour change within professional practice. Evidence from health-related professional fields suggests including post-training activities following training workshops to achieve behaviour change in practice [51,52]. Therefore, future iterations of the DTP intervention should focus on post-training support for driving instructors to facilitate adherence to the intervention protocol. In addition, the completion of manual modules or the dynamic use of the modules (i.e., progression to complex modules for certain driving environments or revisiting of simpler modules for others) was not recorded within this study. The findings highlight the importance of confirming adherence to the protocol and use of the manual modules via fidelity measures in future evaluations of the DTP intervention [9], with the lack of regular fidelity checks a noteworthy limitation in this study. As with most intervention studies, it is possible that selection bias was introduced with the inclusion of highly motivated participants.

## Conclusion

The DTP intervention development was grounded in theories of behaviour modification related to driver education and training addressing challenges faced by autistic student drivers when learning to drive a motor vehicle. The feasibility study provided evidence for preliminary effectiveness and indicated that the intervention was accepted by end-users. This study provided an opportunity to identify and incorporate modifications, refining the DTP for a larger randomised controlled trial.

## Supporting information

S1 File Screening and outcome measures and interview guide used in the study. (PDF)

## References

[1] Vindin P, Wilson NJ, Lee H, Cordier R. The experience of learning to drive for people with autism spectrum disorder. Focus Autism Other Dev Disabl. 2021;36(4):225–36. doi: 10.1177/10883576211023312

[2] Brooks J, Kellett J, Seeanner J, Jenkins C, Buchanan C, Kinsman A, et al. Training the motor aspects of pre-driving skills of young adults with and without autism spectrum disorder. J Autism Dev Disord. 2016;46(7):2408–26. doi: 10.1007/s10803-016-2775-8 27055416

[3] Cox SM, Cox DJ, Kofler MJ, Moncrief MA, Johnson RJ, Lambert AE, et al. Driving simulator performance in novice drivers with autism spectrum disorder: the role of executive functions and basic motor skills. J Autism Dev Disord. 2016;46(4):1379–91. doi: 10.1007/s10803-015-2677-1 26676628

[4] Huang P, Winston FK. Young drivers. Handbook of traffic psychology. Elsevier. 2011. p. 315–38.

[5] Tyler S. Asperger’s Syndrome: The Implications for Driver Training Methods and Road Safety. Journal of the Australasian College of Road Safety. 2013;24(1):55–63.

[6] Lindsay S. Systematic review of factors affecting driving and motor vehicle transportation among people with autism spectrum disorder. Disabil Rehabil. 2017;39(9):837–46. doi: 10.3109/09638288.2016.1161849 27045872

[7] Wilson NJ, Lee HC, Vaz S, Vindin P, Cordier R. Scoping Review of the Driving Behaviour of and Driver Training Programs for People on the Autism Spectrum. Behav Neurol. 2018;2018:6842306. doi: 10.1155/2018/6842306 30245750 PMC6136574

[8] Peräaho M, Keskinen E, Hatakka M. Driver competence in a hierarchical perspective; implications for driver education. Swedish Road Administration. 2003.

[9] Smith T, Scahill L, Dawson G, Guthrie D, Lord C, Odom S, et al. Designing research studies on psychosocial interventions in autism. J Autism Dev Disord. 2007;37(2):354–66. doi: 10.1007/s10803-006-0173-3 16897380

[10] Lai M-C, Anagnostou E, Wiznitzer M, Allison C, Baron-Cohen S. Evidence-based support for autistic people across the lifespan: maximising potential, minimising barriers, and optimising the person-environment fit. Lancet Neurol. 2020;19(5):434–51. doi: 10.1016/S1474-4422(20)30034-X 32142628

[11] Gahan L, Gaffy E, Dow B, Brijnath B. Advancing methodologies to increase end-user engagement with complex interventions: The case of co-designing the Australian elder abuse screening instrument (AuSI). J Elder Abuse Negl. 2019;31(4–5):325–39. doi: 10.1080/08946566.2019.1682098 31647378

[12] Wight D, Wimbush E, Jepson R, Doi L. Six steps in quality intervention development (6SQuID). J Epidemiol Community Health. 2016;70(5):520–5. doi: 10.1136/jech-2015-205952 26573236 PMC4853546

[13] Craig P, Dieppe P, Macintyre S, Michie S, Nazareth I, Petticrew M. Developing and evaluating complex interventions: the new Medical Research Council guidance. BMJ. 2008;337. 10.1136/bmj.a1655 PMC2769032 18824488

[14] Evans D. Hierarchy of evidence: a framework for ranking evidence evaluating healthcare interventions. J Clin Nurs. 2003;12(1):77–84. doi: 10.1046/j.1365-2702.2003.00662.x 12519253

[15] O’Cathain A, Croot L, Sworn K, Duncan E, Rousseau N, Turner K, et al. Taxonomy of approaches to developing interventions to improve health: a systematic methods overview. Pilot Feasibility Stud. 2019;5:41. doi: 10.1186/s40814-019-0425-6 30923626 PMC6419435

[16] Campbell M, Fitzpatrick R, Haines A, Kinmonth AL, Sandercock P, Spiegelhalter D, et al. Framework for design and evaluation of complex interventions to improve health. BMJ. 2000;321(7262):694–6. doi: 10.1136/bmj.321.7262.694 10987780 PMC1118564

[17] Skivington K, Matthews L, Simpson SA, Craig P, Baird J, Blazeby JM, et al. A new framework for developing and evaluating complex interventions: update of Medical Research Council guidance. BMJ. 2021;374:n2061. doi: 10.1136/bmj.n2061 34593508 PMC8482308

[18] Mercier A, Sherrod G, Ennis R, Clay OJ, Richter CG, Stavrinos D. The Driving Profile of Autistic Drivers and Their Driving Experiences: A Systematic Review. J Autism Dev Disord. 2025;55(6):2141–56. doi: 10.1007/s10803-024-06586-x 39395127

[19] Silvi C, Scott-Parker B, Jones C. A Literature Review of the Likely Effects of Autism Spectrum Disorder on Adolescent Driving Abilities. Adolescent Res Rev. 2017;3(4):449–65. doi: 10.1007/s40894-017-0068-x

[20] Hoddinott P. A new era for intervention development studies. Pilot Feasibility Stud. 2015;1:36. doi: 10.1186/s40814-015-0032-0 27965814 PMC5153779

[21] Onken LS, Carroll KM, Shoham V, Cuthbert BN, Riddle M. Reenvisioning Clinical Science: Unifying the Discipline to Improve the Public Health. Clin Psychol Sci. 2014;2(1):22–34. doi: 10.1177/2167702613497932 25821658 PMC4374633

[22] Bartholomew LK. Planning health promotion programs: an intervention mapping approach. 3rd ed. San Francisco, CA: Jossey-Bass. 2011.

[23] Vindin P, Cordier R, Wilson NJ, Lee H. A Driver Training Program Intervention for Student Drivers with Autism Spectrum Disorder: A Multi-site Randomised Controlled Trial. J Autism Dev Disord. 2021;51(10):3707–21. doi: 10.1007/s10803-020-04825-5 33389302

[24] Hatakka M, Keskinen E, Gregersen NP, Glad A, Hernetkoski K. From control of the vehicle to personal self-control; broadening the perspectives to driver education. Transportation Research Part F: Traffic Psychology and Behaviour. 2002;5(3):201–15. doi: 10.1016/s1369-8478(02)00018-9

[25] Michon JA. Dealing with danger. Haren, The Netherlands: Verkeerskundig Studiecentrum, Rijksuniversiteit Groningen. 1979.

[26] Michon JA. A critical view of driver behavior models: What do we know, what should we do? In: Evans L, Schwing RC, Editors. Human behavior and traffic safety. Boston, MA: Springer US. 1985. p. 485–524.

[27] Curry AE, Yerys BE, Huang P, Metzger KB. Longitudinal study of driver licensing rates among adolescents and young adults with autism spectrum disorder. Autism. 2018;22(4):479–88. doi: 10.1177/1362361317699586 28374599 PMC5767541

[28] Gandolfi J. Driver Education - A Blueprint for Success? 2009.

[29] WHO. International classification of functioning, disability and health: ICF. Geneva: World Health Organization. 2001.

[30] Bölte S, Mahdi S, de Vries PJ, Granlund M, Robison JE, Shulman C, et al. The Gestalt of functioning in autism spectrum disorder: Results of the international conference to develop final consensus International Classification of Functioning, Disability and Health core sets. Autism. 2019;23(2):449–67. doi: 10.1177/1362361318755522 29378422 PMC6376609

[31] George S, May E, Crotty M. Exploration of the links between concepts of theoretical driving models and the International Classification of Functioning, Disability, and Health. J Allied Health. 2009;38(2):113–20. 19623793

[32] Winston FK, Mirman JH, Curry AE, Pfeiffer MR, Elliott MR, Durbin DR. Engagement with the TeenDrivingPlan and diversity of teens’ supervised practice driving: lessons for internet-based learner driver interventions. Inj Prev. 2015;21(1):4–9. doi: 10.1136/injuryprev-2014-041212 24916684

[33] Falkmer T, Gustavsson L, Nielsen B, Peters B. Teaching learner drivers with disabilities: An operation manual for driving instructors. Statens väg-och transportforskningsinstitut. 2000.

[34] Mirman JH, Albert WD, Curry AE, Winston FK, Thiel MCF, Durbin DR. TeenDrivingPlan effectiveness: The effect of quantity and diversity of supervised practice on teens’ driving performance. Journal of Adolescent Health. 2014;55(5):620–6. 10.1016/j.jadohealth.2014.04.010 24925492

[35] Bowen DJ, Kreuter M, Spring B, Cofta-Woerpel L, Linnan L, Weiner D, et al. How we design feasibility studies. Am J Prev Med. 2009;36(5):452–7. doi: 10.1016/j.amepre.2009.02.002 19362699 PMC2859314

[36] Precision Vision. Revised 2000 Series ETDRS Charts-- 2 meters: Precision Vision; 2000 [cited 2013 6 May]. Available from: http://precision-vision.com/index.cfm/category/34/etdrs-charts.cfm?CFID=34718261&CFTOKEN=87c96276a5c3005e-78591027-A015-34B9-7B01A7CB930534D5

[37] IBM Corporation. IBM SPSS Statistics for Windows, Version 21.0. Armonk, NY: IBM Corp. 2012.

[38] Cohen J. Statistical power analysis for the behavioral sciences. 2 ed. Hillsdale, NJ: Lawrence Erlbaum Associates, Publishers. 1988.

[39] Kolb SM. Grounded theory and the constant comparative method: valid research strategies for educators. Journal of Emerging Trends in Educational Research and Policy Studies. 2012;3(1):83–6.

[40] Gardner H. Frames of mind: The theory of multiple intelligences. New York: Basic Books. 2011.

[41] Philadelphia TC. Teen Driver Source. The Children’s Hospital of Philadelphia Research Institute. 2018. Available from: https://teendriversource.research.chop.edu/.

[42] Government T. Transport Services 2020 [cited June 2]. Available from: https://www.transport.tas.gov.au/licensing/getting_your_car_licence/learn_to_drive_videos/english

[43] Myers RK, Bonsu JM, Carey ME, Yerys BE, Mollen CJ, Curry AE. Teaching Autistic Adolescents and Young Adults to Drive: Perspectives of Specialized Driving Instructors. Autism Adulthood. 2019;1(3):202–9. doi: 10.1089/aut.2018.0054 32292888 PMC6745536

[44] Myers RK, Carey ME, Bonsu JM, Yerys BE, Mollen CJ, Curry AE. Behind the wheel: Specialized driving instructors’ experiences and strategies for teaching autistic adolescents to drive. American Journal of Occupational Therapy. 2021;75(3):7503180110p1–p11. 10.5014/ajot.2021.043406 PMC8095704 34781345

[45] Bates L, Hawkins A, Rodwell D, Anderson L, Watson B, Filtness AJ, et al. The effect of psychosocial factors on perceptions of driver education using the goals for driver education framework. Transportation Research Part F: Traffic Psychology and Behaviour. 2019;66:151–61.

[46] Molina JG, García-Ros R, Keskinen E. Implementation of the driver training curriculum in Spain: An analysis based on the Goals for Driver Education (GDE) framework. Transportation Research Part F: Traffic Psychology and Behaviour. 2014;26:28–37. doi: 10.1016/j.trf.2014.06.005

[47] Rodwell D, Hawkins A, Haworth N, Larue GS, Bates L, Filtness A. A mixed-methods study of driver education informed by the Goals for Driver Education: Do young drivers and educators agree on what was taught?. Safety science. 2018;108.

[48] NDIS. Will you be driving the vehicle? [cited 2023 April 19]. Available from: https://ourguidelines.ndis.gov.au/supports-you-can-access-menu/equipment-and-technology/vehicle-modifications-and-driving-supports/how-do-you-get-vehicle-modifications-and-driving-supports-your-plan/will-you-be-driving-vehicle

[49] Brown HM, Oram-Cardy J, Johnson A. A meta-analysis of the reading comprehension skills of individuals on the autism spectrum. J Autism Dev Disord. 2013;43(4):932–55. doi: 10.1007/s10803-012-1638-1 23054199

[50] Curry AE, Peek-Asa C, Hamann CJ, Mirman JH. Effectiveness of Parent-Focused Interventions to Increase Teen Driver Safety: A Critical Review. J Adolesc Health. 2015;57(1 Suppl):S6-14. doi: 10.1016/j.jadohealth.2015.01.003 26112737 PMC4483193

[51] Forsetlund L, O’Brien MA, Forsén L, Reinar LM, Okwen MP, Horsley T, et al. Continuing education meetings and workshops: effects on professional practice and healthcare outcomes. Cochrane Database Syst Rev. 2021;9(9):CD003030. doi: 10.1002/14651858.CD003030.pub3 34523128 PMC8441047

[52] Frank HE, Becker-Haimes EM, Kendall PC. Therapist training in evidence-based interventions for mental health: A systematic review of training approaches and outcomes. Clin Psychol (New York). 2020;27(3):e12330. doi: 10.1111/cpsp.12330 34092941 PMC8174802

