# Supplementary material for: Development and feasibility of a driving training program for Autistic student drivers
Source: PLoS One. 2025 Jun 27;20(6):e0324934. doi: 10.1371/journal.pone.0324934 (PMC12204471; doi:10.1371/journal.pone.0324934)
Supplement: S1 File — (PDF) [file pone.0324934.s001.pdf]

Screening and outcome measures and interview guide used in the study

Screening measures

Behaviour Rating Inventory of Executive Function Adult version (BRIEF-A)

A self- and informant-report measure of executive function and self-regulation in the individual's everyday environment. The measure comprises 75 items with eight subscales: inhibit, shift, emotional control (behavioural regulation scales), initiate, self-monitor, working memory, plan/organise, and task monitor (metacognition scales). The BRIEF-A has high internal consistency (Cronbach's α = 0.80-0.98), moderate inter-rater reliability (Pearson's r = 0.32) and high test-retest reliability (r = 0.82; Gioia et al., 2002).

Cambridge Neuropsychological Test Automated Battery (CANTAB)

A measure of executive function assessing planning, episodic memory and processing speed. Five subscales were chosen to measure aspects of executive function relevant to autism and driving: visual memory (paired associates learning), working memory (spatial working memory), planning efficiency (one-touch stockings of Cambridge), processing speed (reaction time), and managing conflicting and task-irrelevant information (multi-tasking test). The CANTAB subtests have high internal consistency (Ozonoff et al., 2004).

Barratt Impulsiveness Scale (BIS-II)

A measure of impulsivity containing 30 self-report items, answered on a 4-point Likert scale. The BIS-II assesses attentional (attention and cognitive instability), lack of

planning (self-control and cognitive complexity), and motor impulsivity (motor and perseverance) and has high test-retest reliability and internal consistency (Cronbach's α = 0.83; Patton et al., 1995).

Social Responsiveness Scale (SRS-2)

The SRS-2 measure the level of social support needs of student drivers via five subscales: social awareness, social cognition, social communication, social motivation and autistic mannerisms. The SRS-2 has high internal consistency (Chronbach's α = 0.91-0.97; Bölte, 2012). A total score of 76 or higher is considered to be associated with autism diagnosis, and scores of 66-75 indicate high to moderate support needs in reciprocal social behaviour and may be indicative of autism (Constantino & Gruber, 2005). The total SRS-2 scores for participants in this study were ≥71, consistent with an autism diagnosis and indicating moderate to high social support needs.

Revised Children's Manifest Anxiety Scale, second edition (RCMAS-2)

A self-report measure of trait anxiety across five domains: defensiveness, inconsistent responding, worry, and physiological and social anxiety. The RCMAS-2 contains 49 items with a 'yes' or 'no' response format to measure overall and performance anxiety (Reynolds & Richmond, 2008). The RCMAS-2 has high internal consistency (Cronbach's α = 0.75-0.92; Ambler et al., 2015).

Adelaide Driving Self-Efficacy Scale (ADSES)

A self-report measure of driving ability self-efficacy. Participants rated their confidence (0 = not confident, 10 = completely confident) against 12 driving behaviours. The ADSES has high internal consistency (Cronbach's α = 0.98; George et al., 2007).

NASA Task Load Index (NASA TLX).

A self-report measure of perceived mental workload comprises six dimensions: mental demand, physical demand, temporal demand, performance, effort, and frustration (Hart, 2006).

Outcome measure

Driving Performance Checklist (DPC).

The DPC is an observational measure of on-road driving performance, using an activity analysis approach to score driving performance by breaking driving checkpoints into specific tasks (Chee et al., 2017; Lee et al., 2003). For example, at a T-intersection (left-turn), the student driver is required to complete the following steps: 1) signal left, 2) decelerate when approaching the sign, 3) check oncoming traffic (head turns), and 4) proceed when way is clear. A trained graduate research assistant observed and rated each participant’s driving performance while seated directly behind the passenger seat. One point was given for each step correctly performed, with the total scores summed for each driving checkpoint. A combination of seven left-turn T-junctions, seven right-turn T-junctions, eight roundabouts, six traffic light intersections and two pedestrian areas were included in the rating (Chee et al., 2017).

Interview guide

An interview guide was developed to evaluate the feasibility of the DTP intervention from the end-user's perspective. The guide consisted of open-ended questions on the following topics: (a) experience taking part in the intervention, (b) driving training effectiveness, and (c) program components use and logistics. At the end of the interviews, participants were asked to rate their agreement with a series of statements about their or the

student driver’s experience, confidence, benefits, and communication with the research team. Participants rated their responses on a 10-point scale (1 = ‘I don’t agree at all’ to 10 = ‘I totally agree’). The final question required participants to rate the program's usability (driving lessons, manual and learning log) on a 10-point scale (1 = ‘very difficult’ to 10 = ‘very easy’.

References

Ambler, P. G., Eidels, A., & Gregory, C. (2015). Anxiety and aggression in adolescents with autism spectrum disorders attending mainstream schools. *Research in Autism Spectrum Disorders*, *18*, 97-109. https://doi.org/10.1016/j.rasd.2015.07.005

Bölte, S. (2012). Brief Report: The Social Responsiveness Scale for Adults (SRS-A): Initial Results in a German Cohort. *Journal of Autism and Developmental Disorders*, *42*(9), 1998-1999. https://doi.org/10.1007/s10803-011-1424-5

Chee, D. Y., Lee, H. C., Patomella, A.-H., & Falkmer, T. (2017). Driving behaviour profile of drivers with autism spectrum disorder (ASD). *Journal of Autism and Developmental Disorders*, *47*, 2658-2670. https://doi.org/10.1007/s10803-017-3178-1

Constantino, J. N., & Gruber, C. P. (2005). *Social Responsiveness Scale (SRS) Manual*. Western Psychological Services.

George, S., Clark, M., & Crotty, M. (2007). Development of the Adelaide Driving Self-Efficacy Scale. *Clinical Rehabilitation*, *21*(1), 56-61. https://doi.org/10.1177/0269215506071284

Gioia, G. A., Isquith, P. K., Retzlaff, P. D., & Espy, K. A. (2002). Confirmatory Factor Analysis of the Behavior Rating Inventory of Executive Function (BRIEF) in a Clinical Sample. *Child Neuropsychology*, *8*(4), 249-257. https://doi.org/10.1076/chin.8.4.249.13513

Hart, S. G. (2006). Nasa-Task Load Index (NASA-TLX); 20 Years Later. *Proceedings of the Human Factors and Ergonomics Society Annual Meeting*, *50*(9), 904-908. https://doi.org/10.1177/154193120605000909

Lee, H. C., Cameron, D., & Lee, A. H. (2003). Assessing the driving performance of older adult drivers: On-road versus simulated driving. *Accident Analysis & Prevention*,

35(5), 797-803. https://doi.org/10.1016/S0001-4575(02)00083-0

Ozonoff, S., Cook, I., Coon, H., Dawson, G., Joseph, R. M., Klin, A., McMahon, W. M., Minshew, N., Munson, J. A., Pennington, B. F., Rogers, S. J., Spence, M. A., Tager-Flusberg, H., Volkmar, F. R., & Wrathall, D. (2004). Performance on Cambridge Neuropsychological Test Automated Battery Subtests Sensitive to Frontal Lobe Function in People with Autistic Disorder: Evidence from the Collaborative Programs of Excellence in Autism Network. *Journal of Autism and Developmental Disorders*, *34*(2), 139-150. https://doi.org/10.1023/B:JADD.0000022605.81989.cc

Patton, J. H., Stanford, M. S., & Barratt, E. S. (1995). Factor structure of the barratt impulsiveness scale. *Journal of Clinical Psychology*, *51*(6), 768-774. https://doi.org/https://doi.org/10.1002/1097-4679(199511)51:6<768::AID-JCLP2270510607>3.0.CO;2-1

Reynolds, C. R., & Richmond, B. O. (2008). *Revised Children's Manifest Anxiety Scale–Second Edition (RCMAS-2)*. Western Psychological Services.
